# Numerical Analysis of Steel Geodesic Dome under Seismic Excitations

**DOI:** 10.3390/ma14164493

**Published:** 2021-08-10

**Authors:** Dominika Bysiec, Tomasz Maleska

**Affiliations:** Faculty of Civil Engineering and Architecture, Opole University of Technology, 45-758 Opole, Poland; d.bysiec@po.edu.pl

**Keywords:** geodesic dome, seismic response, dynamic analysis, seismic analysis

## Abstract

The paper presents the response of two geodesic domes under seismic excitations. The structures subjected to seismic analysis were created by two different methods of subdividing spherical triangles (the original octahedron face), as proposed by Fuliński. These structures are characterised by the similar number of elements. The structures are made of steel, which is a material that undoubtedly gives lightness to structures and allows large spans. Designing steel domes is currently a challenge for constructors, as well as architects, who take into account their aesthetic considerations. The analysis was carried out using the finite element method of the numerical program. The two designed domes were analysed using four different seismic excitations. The analysis shows what influence particular earthquakes have on the geodesic dome structures by two different methods. The study analysed the maximum displacements, axial forces, velocities, and accelerations of the designed domes. In addition, the Time History method was used for the analysis, which enabled the analysis of the structure in the time domain. The study will be helpful in designing new structures in seismic areas and in assessing the strength of various geodesic dome structures under seismic excitation.

## 1. Introduction

Strut domes are an effective two-curved cover, which is approximated by a mesh of struts. The use of less material and lower costs, combined with the possibility of covering very large areas, is a definite advantage of this type of structure. The stiffness of these three-dimensional systems justifies their ability to cover large spans with a small amount of construction material. Architects and engineers all over the world use a wide range of possibilities of connecting strut elements, constituting a mesh of dome covers. The aesthetic potential of this engineering system means that the structure has a valuable architectural aspect [1]. This aspect was important not only in steel geodesic domes but also in other spatial structures (e.g., made of concrete) [2].

From dome ceilings and full buildings to Arctic homes and artificial biomes, geodesic domes around the world continue to inspire and amaze both architecture enthusiasts and curious travellers. The elegant and aerodynamic form of geodesic domes creates expansive yet economical spaces that are ideal for greenhouses, arenas, sports facilities, entertainment halls, swimming pools, and other uses. For centuries, dome structures were used due to their thermal advantages, structural benefits, and availability of construction materials [1].

After Fuller [1] patented methods of dividing a sphere into spherical triangles in 1954 (based on an icosahedron as the initial solid), steel mesh domes of the geodetic type have almost completely replaced the use of other types of domes. In 1967, his design was shown to the world as a ‘Biosphere’, with a diameter of 76 m, constructed for Expo ‘67 in Montreal.

Fuller [1] believed that the geodesic dome was nature’s perfect structure, enclosing the greatest space with the least amount of material. While remaining in tune with the environment, the dome supports itself without the need for any internal columns or walls. The largest geodesic dome projects include the Fukuoka Dome (built in Fukuoka, Japan in 1993, 216 m), Nagoya Dome (Nagoya, Japan in 1997, 187 m), Louvre (Abu Dhabi, United Arab Emirates, 180 m), Tacoma Dome (Tacoma, WA, USA, 161 m), and the Superior Dome (Northern Michigan University, Marquette, MI, USA, 160 m).

The advantage of Fuller’s geodesic strut domes is the small number of struts required at different lengths. According to his patent, the domes were formed on the basis of the icosahedron, which requires the smallest number of groups of struts of equal lengths. There were many papers related to such geodesic domes. Significant and excellent achievements in lightweight, durable, and self-supporting structures have been attained by Makowski [3,4], Clinton [5,6], Tarnai [7,8,9], Huybers [10,11,12,13,14,15], Lalvani [16,17,18,19], Pavlov [20,21], Ramaswamy [22], Obrębski [23], Szmit [24,25], and Rębielak [26].

Although dome structures are economical in terms of consumption of construction materials compared to the conventional forms of structures [27], a more lightweight design can be obtained using optimisation methods. The optimum solution of the geometry design was obtained by Saka [28], Kaveh and Talatahari [29], Carvalho et al. [30], Saka and Carbas [31], Gholizadeh and Barati [32], Kaveh and Rezaei [33,34], Kaveh et al. [35], and Ye and Lu [36]. Other structures were analysed from the economical aspect, e.g., bridge [37,38], but in these cases, the methods and reasons of optimisation were totally different.

An analysis of the behaviour of shallow geodesic lattice domes was presented by Guan et al. [39]. Barbieri et al. [40] analysed the dynamic behaviour of a geodesic dome in aluminium alloy through numerical models obtained using the finite element method. Szaniec and Zielinska [41] presented the results of a dynamic analysis of an existing reticulated dome under wind loads. The calculation model of the structure was constructed using the finite element method. The dome was subjected to the standard wind pressure, assuming that it operates harmonically. Satria et al. [42] considered the dynamic behaviour of a new type of two-way single layer lattice dome with nodal eccentricity. Fu [43] presented a static analysis and design of tensegrity domes. New forms of the tensegrity domes were proposed.

Studies of geodesic domes under seismic loads have rarely been investigated. A few papers on this subject can be found. In the paper of Cai et al. [44], the dynamic characteristics of a space beam string structure was determined. The aim of the paper was to determine the structure’s response to seismic excitation using the Time History method and the modal method. These methods were used to determine the structure’s response to the given seismic excitation. In the paper by Takeuchi et al. [45], the response evaluation method of domes and cylindrical shell roofs with substructures was shown. In addition, the response amplification factors approach was proposed. Furthermore, Nakazawa et al. [46] focused on methods for evaluating responses under seismic loads to metal roof spatial structures. In addition, papers by Kato and Nakazawa [47], Li et al. [48], and Qin et al. [49] focused on the assessment of lightweight structure responses exposed to earthquakes. In addition, the dynamic stability and failure probability analysis of dome structures under stochastic seismic excitation was presented by Li and Xu [50].

It should be added that the most of the analysis on geodesic domes relate to space frames, the basis of which is the icosahedron, which is the development of Fuller’s patent. Davis [51] showed that the octahedron might seem to be a better option for geodesic domes than the icosahedron. However, the problems arise because more subdivisions were required, and thus, more different lengths were required.

The paper presents the numerical analysis of the geodesic domes under seismic excitations. The developed structures were created on the basis of the regular octahedron, which was a polyhedron that has not been considered in great detail so far [52,53,54]. Two different methods for the subdivision of the spherical triangle proposed by Fuliński [1,55] were used to design the two geodesic domes under seismic analysis. The numerical analyses were carried out for four different seismic excitations. Different times and intensities of the seismic records were presented. The results obtained from numerical analysis were compared with two structures differing in subdivision methods and under four different excitations. The presented analysis was the first step for further consideration of the optimisation of the geodesic dome. It is very important to take into account the wider possibilities of using this type of structure to cover large areas. It should be added that the geodesic domes around the world continue to inspire and amaze both architectural enthusiasts and curious travellers.

## 2. Description of Numerical Modelling

### 2.1. Subdivision Methods for Spherical Dome (Strut) Structures Based on the Regular Octahedron

**Figure 1:**
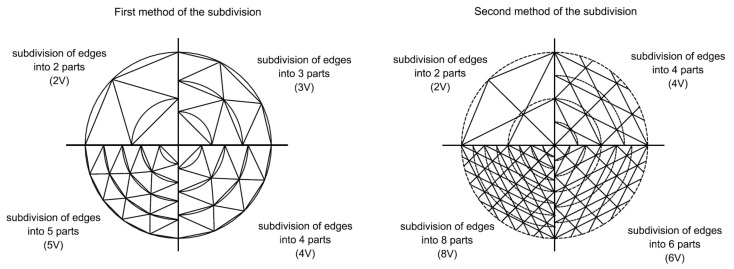
Methods of subdividing the initial triangle edge, according to Fuliński.

The two developed geodesic domes under the seismic excitations were shaped in accordance with two methods proposed by Fuliński [55]. The structures were designed on the basis of being regular octahedra, subdividing their equilateral faces into smaller sub-faces and taking the resulting face vertices to define the nodes of the structural grid, while the edges of the sub-faces define the axes of the structural members. Both of the methods used lead to the division of the initial triangle of the octahedron into smaller triangles by frequency (V), i.e., the number of subdivisions. The subdivision process naturally leads to the generation of a three-way grid on every face of the basis octahedron. The central projection of this grid’s vertices on the octahedron’s circumscribed sphere leads to a polyhedron approximating the sphere in which only the grid’s nodes lie on the sphere’s surface. More parts give smoother spheres. The mentioned methods were developed in detail in the paper by Pilarska [54]. Figure 1 shows the difference in the shaping of geodesic domes using the first and second subdivision methods.

### 2.2. Tested Models

**Figure 2:**
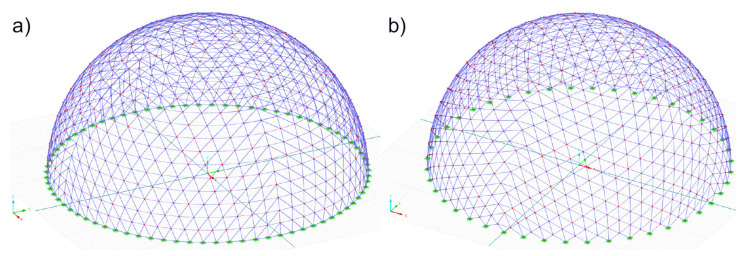
View of numerical model: (**a**) method 1 and (**b**) method 2.

Two geodesic strut domes were subjected to numerical analysis, taking into account the seismic excitations. These structures were designed according to the two different methods of creating geodesic domes proposed by Fuliński [55], which are presented in Section 2.1 and described in detail in the papers by Pilarska [52,53,54]. The basis for generating the meshes of both structures was a regular octahedron. It was an initial triangle and was divided into as many parts to finally obtain domes characterised by a similar number of struts. Including the first method, after dividing the initial triangle of the regular octahedron into 19 parts, 2888 hedra were obtained, i.e., a structure consisting of 761 nodes and of 2204 struts (Figure 2a). The analysed dome was 49.97 m wide and 25.0 m high. Using the second method, after dividing the initial triangle of the regular octahedron into 22 parts, 2904 hedra were modelled. This dome consists of 749 nodes and 2156 struts (Figure 2b), with a width of 50.0 m and a height of 25.0 m.

### 2.3. Material of Numerical Models

The analysed geodesic domes were made of steel struts (round pipes) of S235 standard steel. The properties of this steel grade were: (i) Young modulus (*E*) 210 GPa, (ii) Kirchhoff module (*G*) 80.76 GPa, (iii) Poisson ratio (*ν*) 0.3, (iv) volumetric weight (*γ*) 7850 kg/m^3^, (v) thermal expansion coefficient (*α*) 1.2 × 10^−5^, and (vi) partial safety factor (*γ_M_*) 1.0. The steel elements were modelled as a linearly elastic isotropic material (according to Eurocode 3 [56]). All of the struts in both analysed domes were grouped into four groups, taking into account a different cross-section (commonly used cross-section) of the elements, as shown in Table 1.

### 2.4. Description of Numerical Analysis

The DLUBAL RFEM 5.21.01 (2020, Dlubal Software GmbH from Tiefenbach, Germany) numerical program was used to analyse the effect of seismic excitations of the geodesic domes. This program is based on the finite element method. This method enables the determination of the maximum value (e.g., displacement or acceleration) in a given node of a finite element. The mentioned numerical program (RFEM) is widely used by engineers all over the world. It gained its popularity thanks to extensive specialised modules. In this paper, the RF-DYNAM PRO 5.21.01 (2020, Dlubal Software GmbH from Tiefenbach, Germany) module was used for dynamic analysis of the structure response. In the analysed numerical models, the size of the finite elements did not exceed of 0.2 m. The supports of dome were modelled as rigid. The purpose of such modelling of the supports was to obtain the maximum response of the dome structure to the given excitation. This proposition of support was commonly used in engineering practice, e.g., by Chmielewski et al. [57], Tabatabaiefar and Massumi [58]. In a geodesic dome, the Soil–Structure Interaction (SSI) effect can be omitted, because in these structures, there is no effect of: (i) second order, (ii) massive, (iii) slender tall structure, (iv) or soft soil from Eurocode 8 [59].

### 2.5. Seismic Excitation

**Figure 3:**
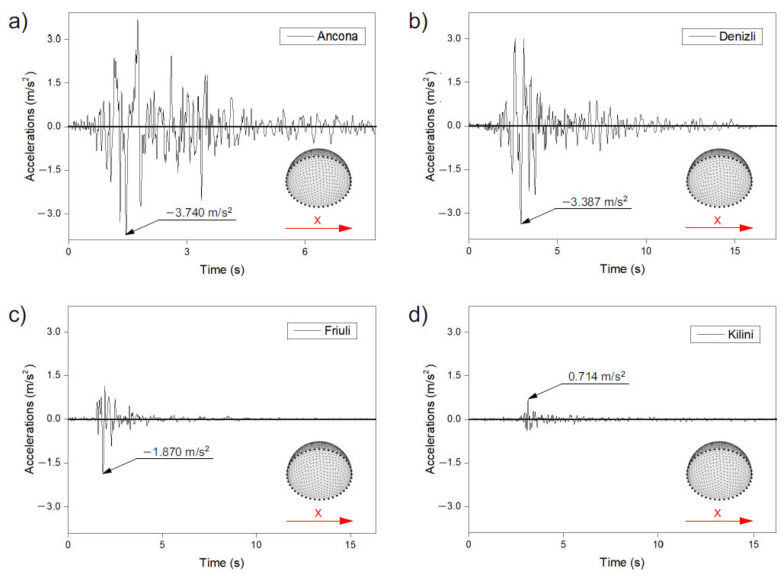
The most intensive component of seismic excitations (x-direction): (**a**) Ancona (Italia), (**b**) Denizli (Turkey), (**c**) Friuli (Italia), (**d**) Kilini (Greece).

In order to assess the effect of seismic response on geodesic domes, four different seismic records were used, i.e., (i) Ancona, (ii) Denizli, (iii) Friuli, and (iv) Kilini. The Ancona record (Italy) comes from the Genio-Civile station on June 14, 1972. It was characterised by ground acceleration equal to −3.740 m/s^2^ (Figure 3a) and duration of 7.76 s. The second record Denizli (Turkey) was recorded at the station Denizli-Bayindirlik ve Iskan Mudurlugu and dates from August 19, 1976. For this recording, the ground acceleration value was −3.387 m/s^2^ (Figure 3b); this recording lasted 17.31 s. Another record came from Italy (Friuli), exactly from the Somplago-Uscita Galleria station on September 16, 1977. The maximum value of ground acceleration during this earthquake was −1.870 m/s^2^ (Figure 3c) and lasted 16.30 s. The last weakest record came from Kilini (Greece) at Vartholomio Residence station on October of 31, 1998, and its maximum ground acceleration was 0.714m/s^2^ (Figure 3d) in 16.18 s.

## 3. Result of Numerical Analysis

### 3.1. Method of Numerical Analysis

The study assesses the effect of seismic excitations on the geodesic domes, which were designed in accordance with two different methods for creating these types of structures. As mentioned earlier, these methods have been described in detail in the papers [52,53,54]. The numerical program RFEM and the Time History method were used for the analysis. This method allows for the analysis of the structure in given time steps. The time step of numerical analysis was equal to 0.02 s. The load was applied in accordance with the standard rules [59], i.e., in two horizontal directions (X and Y) and in the vertical direction (Z). In addition, the seismic excitations were applied simultaneously in all direction (direction of X, Y and Z) to the rigid supports.

Table 1 shows the results for the analysed domes under seismic excitations. The analysed records had different durations and intensities. The dynamic (seismic) analysis was carried out on the basis of the numerical models presented in [52,53,54]. The above-mentioned publications applied the rule that the most stressed strut in a given group was at a level of 90%. For the purposes of analysing the obtained results, eight numerical models were created, by different structural methods (method 1 or method 2). In addition, the seismic excitations (Ancona, Denizil, Friuli, and Kilini) were used. As a result, eight models were obtained, i.e., model I (method 1, Ancona), model II (method 2, Ancona), model III (method 1, Denizil), model IV (method 2, Denizil), model V (method 1, Friuli), model VI (method 2, Friuli), model VII (method 1, Kilini), and model VIII (method 2, Kilini).

### 3.2. Displacements

Based on the analysis, it can be seen that the maximum displacement values were recorded for the horizontal directions X and Y (for the Ancona and Denzil records in the Y direction, for the Friuli and Kilini records in the X direction). In the vertical direction, the displacements were much smaller (Table 2). For all four of the seismic records analysed, higher values were obtained for the dome designed according to method 1 than for the dome generated according to method 2.

**Figure 4:**
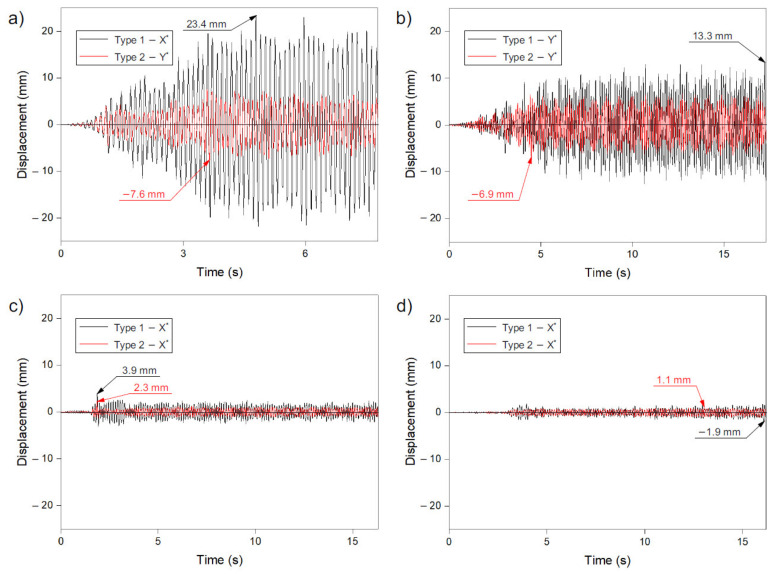
Maximum displacements in numerical model records: (**a**) Ancona—models I and II, (**b**) Denizli—models III and IV, (**c**) Friuli—models V and VI, (**d**) Kilini—models VII and VIII.

In the case of structures shaped on the basis of method 1, the highest displacement values were obtained with the Ancona record (model I, 23.4 mm; Figure 4a). In the case of other records, the displacement values were smaller. In relation to Ancona, they accounted for 56% for Denizli (model III, 13.3 mm; Figure 4b), 17% for Friuli (model V, 3.9 mm; Figure 4c), and 8% for Kilini (model VII, −1.9 mm; Figure 4d). A similar tendency was noticeable with the domes created according to method 2. The Ancona forcing caused the highest displacement values (model II, −7.6 mm; Figure 4a), while for the remaining records, the maximum displacement values were 90% (model IV, Denizli, −6.9 mm; Figure 4b), 30% (model VI, Friuli, 2.3 mm; Figure 4c) and 14% (model VIII, Kilini, 1.1 mm; Figure 4d) of the values from the Ancon record, respectively.

There was also a noticeable difference in the size of the maximum displacements between the structures shaped according to methods 1 and 2. The greatest differences were obtained for the Ancona record, in which the dome shaped according to method 1 showed about three times greater maximum displacements (model I, 23.4 mm; Figure 4a) than the dome designed according to method 2 (model II, 7.6 mm; Figure 4b). In the case of the records of Denizli, Friuli, and Kilini, the differences were about two times greater for method 1 compared to method 2.

It should be added that the value of displacements for the analysed numerical models decreases with a decrease in the value of ground acceleration, which was recorded for individual earthquake records. The highest values were obtained for the Ancona record, i.e., the record with the greatest ground acceleration (3.740 m/s^2^).

It can also be seen that the domes were excited for approximately 1.5 to 4.0 s of the recording duration as a result of the imposed excitation. In the first stages of the excitation, the displacements had a negligible value, which was undoubtedly influenced by the characteristics of the given excitation (in the first seconds of recording, the acceleration values were low). However, after the occurrence of the maximum acceleration in a given record (Ancona, Denizli, Friuli, Kilini), the recorded displacements had a similar value to the maximum ones. This tendency was noticeable for all analysed records, regardless of the direction of the excitation. Even the decrease in acceleration did not cause any significant reduction in the displacements of the analysed domes. For both of the domes created according to methods 1 and 2, it can be seen that the moments of occurrence of the maximum values of displacement in the domes were similar to each other. They occurred at very similar time intervals. Only when recording Denizli (model III and model IV) did the maximum displacements appear at significant time intervals: for method 1 (model III) around 17 s and, for method 2 (model IV), approximately 5 s.

### 3.3. Axial Forces

Based on the numerical analysis, it can be concluded that the maximum axial forces occurring in the struts in both of the analysed methods of shaping geodetic strut domes (methods 1 and 2) were similar for each given excitation. Thus, there was no clear trend as in the case of displacements where, for the analysed models built according to method 1, the maximum values of displacement were always higher than for the models shaped according to method 2. It was noticed that for high-intensity records (Ancona and Denizli excitations—Figure 3a,b), higher values of axial forces were obtained for models I and III, where method 1 was used to generate the structure. However, in the case of records of lower intensity (Friuli and Kilini—Figure 3c,d), higher values were recorded for the dome designed according to method 2 (models VI and VIII). The difference in the maximum values of the axial forces of the domes generated according to methods 1 and 2, for the four records analysed, was from 4% to 32% and was largest (32%) was for the Ancona record.

**Figure 5:**
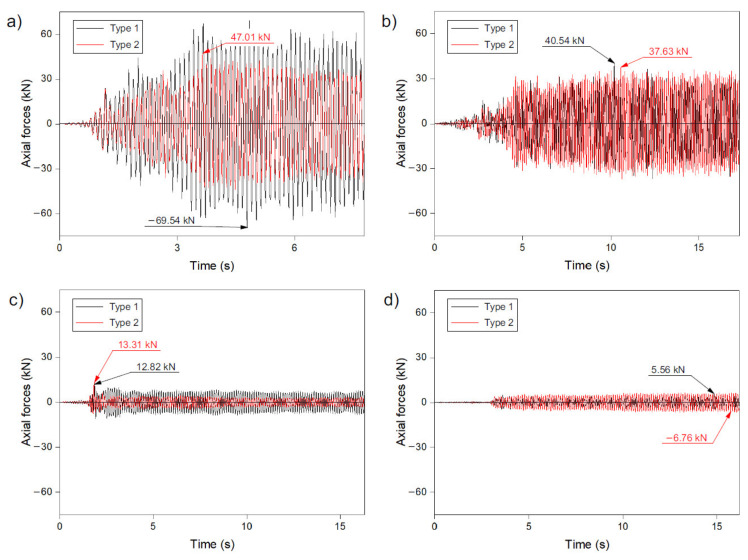
Maximum axial forces in numerical model records of: (**a**) Ancona—models I and II, (**b**) Denizli—models III and IV, (**c**) Friuli—models V and VI, (**d**) Kilini—models VII and VIII.

The greatest forces were recorded with the Ancon excitation in model I (−69.54 kN—Figure 5a), which was designed according to method 1. In the other models, the values were lower and amounted to: (i) 47.01 kN (method 2, model II, Ancona—Figure 5a), (ii) 40.54 kN (method 1, model III, Denizli—Figure 5b), (iii) 37.63 kN (method 2, model IV, Denizli—Figure 5b), (iv) 13.31 kN (method 2, model VI, Friuli—Figure 5c), (v) 12.82 kN (method 1, model V, Friuli—Figure 5c), (vi) −6.76 kN (method 2, model VIII, Kilini—Figure 5d), and (vii) 5.56 kN (method 1, model VII, Kilini—Figure 5d). On the basis of the obtained results presented in Figure 5, it can be seen that the tensile and compression forces in the struts were of a similar value. In some models, almost identical values for the compressive and tensile forces were obtained.

It can also be seen that the axial forces for all analysed models (I–VIII) were recorded after the appearance of the maximum acceleration in the record. From that moment, they remained at relatively equal levels, similar to the displacements. Concentrating on Figure 5, it can also be seen that the maximum forces (for method 1 and 2 of strut dome design) appeared at a similar time. Thus, it can be concluded that the method of shaping the dome structure does not have a significant impact on the moment of the maximum axial forces in the structure appearance. Moreover, no clear tendency can be drawn as to how the length of the records and their intensity impacts the appearance of maximum values over time. As can be seen in Figure 5, the location of the maximum axial forces occurrence in time was very varied.

### 3.4. Velocity

**Figure 6:**
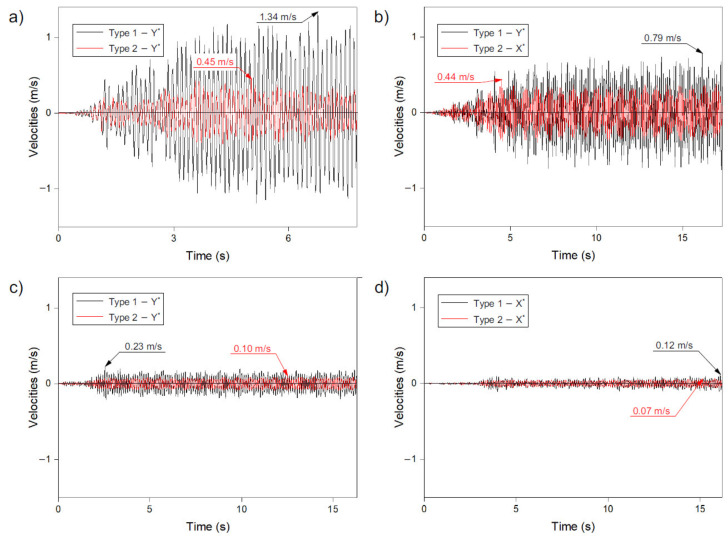
Maximum velocities in numerical model record of: (**a**) Ancona—models I and II, (**b**) Denizli—models III and IV, (**c**) Friuli—models V and VI, (**d**) Kilini—models VII and VIII.

Analysing the maximum velocities, the impact of two different methods of creating strut dome structures can be observed. Based on the obtained results, it can be seen that using method 1 for the construction of a dome, the vibration of velocities as a result of the seismic excitation was much higher than for the structures formed according to method 2 (Figure 6). This tendency was noted in both the records with high ground acceleration (Ancona, Denizli) and records with low ground acceleration (Friuli, Kilini).

In the analysed numerical models (models I–VIII), the highest values for velocity were obtained in model I, i.e., in the dome created according to method 1, with the Ancona record. This value was 1.34 m/s (Figure 6a). In the other models, lower values, ranging from 41% to 95%, (compared to model I) were obtained.

It should also be mentioned that there was a slight difference between the velocities from models II and IV (method 2), which was only 2%. This was not much, considering the fact that for the same excitations in the case of method 1, the difference between models I and III was 41%.

On the basis of Figure 6, it can be seen that the places of the occurrence of the maximum velocities in time for individual records and methods of shaping the dome structure were different. Therefore, it was not possible to clearly define the influence of the acceleration value of the analysed recording as well as the method of shaping the dome (methods 1 or 2). On the other hand, it can be seen (especially in the case of domes designed according to method 1) that as the record intensity decreases, the velocity values decrease. For method 2, it was less noticeable because the obtained results were lower in the range of 42% to 66% (compared to method 1).

However, it can be seen that the strut domes were excited as a result of the seismic excitation application. Figure 6 shows that after the maximum ground acceleration occurs in the record (Figure 3), the vibration velocities remain at relatively the same level for a particular record for the rest of its duration. The method of shaping (method 1 or 2) of the strut domes does not matter.

### 3.5. Acceleration

**Figure 7:**
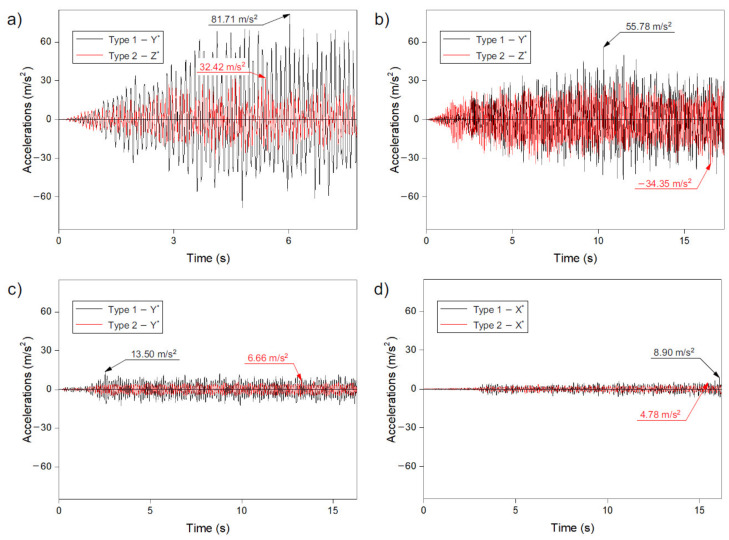
Maximum accelerations in numerical model record of: (**a**) Ancona—models I and II, (**b**) Denizli—models III and IV, (**c**) Friuli—models V and VI, (**d**) Kilini—models VII and VIII.

In the case of maximum accelerations, a similar tendency can be observed for displacements, axial forces, and velocities. The method of generating the strut dome structure clearly matters. For method 1, higher values were obtained than for method 2. This trend was repeated for all analysed records, i.e., Ancona, Denizli, Friuli, and Kilini (Figure 7).

It was noticed that with a decrease in the intensity of the excitation, the maximum accelerations of the dome structure were reduced. Thus, it can be concluded that for the accelerations of the domes, the ground acceleration value has greater significance than the duration of the earthquake.

The highest accelerations were observed in model I (method 1, Ancona), where the obtained value was 81.71 m/s^2^ (Figure 7a). In the other analysed models, the acceleration values in the dome were lower compared to model I and amounted to (i) 55.78 m/s^2^ (method 1, model III, Denizil—Figure 7b), (ii) −34.35 m/s^2^ (method 2, model IV, Denizil—Figure 7b), (iii) 32.42 m/s^2^ (method 2, model II, Ancona—Figure 7a), (iv) 13.50 m/s^2^ (method 1, model V, Friuli—Figure 7c), (v) 8.90 m/s^2^ (method 1, model VII, Kilini—Figure 7d), (vi) 6.66 m/s^2^ (method 2, model VI, Friuli—Figure 7c) and (vii) 4.78 m/s^2^ (method 2, model VIII, Kilini—Figure 7d). Analysing the results of the obtained values, it should be also noted that in the case of domes created according to method 2, higher values were obtained in model IV (Denizil) than in model II (Ancona), although the value of ground acceleration for the given excitation in model II was higher by 10%.

Taking into account the time of the appearance of the maximum accelerations in the dome, it can be seen that it was not identical for the analysed methods of creating the dome (methods 1 and 2). In models I and II, as well as VII and VIII, the time when the maximum values appeared was similar. On the other hand, in models III, IV, V, and VI, big differences were noted between the time when the maximum acceleration values appeared in the dome. The difference was 10 s (V and VI models), which was a very significant value with the recording duration of about 17 s.

As in the case of the previously analysed quantities (displacements, axial forces, and velocities), it was noticed that after the occurrence of the maximum input in the record, the structure was excited. From that moment, the acceleration values were relatively close to the maximum values.

## 4. Conclusions

After the numerical analysis, it can be concluded that the method of shaping the steel structure of domes (methods 1 and 2) has a significant impact on the obtained values of displacements, axial forces, velocities, and accelerations. In addition, the following were noted:

- Displacements in strut domes, constructed according to method 1, were much greater than for domes constructed according to method 2. This tendency was repeatable for high and low intensity records;
- The length of the earthquake record was not significant. The value of the ground acceleration recorded during the earthquake seems more important;
- After the occurrence of the maximum ground acceleration in the analysed record, the steel structure of the cover was excited (regardless of the method of shaping the dome). After excitation of the structure, for the rest of the record, the values remain near the maximum values recorded in the given record,
- The maximum values were mainly obtained in the horizontal directions (X and Y). Only the maximum accelerations for models II and IV, generated according to method 2, were obtained in the vertical direction Z. This shows a close relationship between the shape of the dome structure and their seismic response.

Undoubtedly, the numerical analysis that was performed allowed the determination of how seismic excitation affects the dome shaping method. However, the obtained results were carried out only on relatively small domes. They were new structures, without damage, which was also important. In future, additional analyses are planned for domes of different sizes, as well as damaged domes. In addition, it is planned to optimise the dome structure under seismic excitation.

The obtained results may be helpful in designing this type of structure in seismic areas and may also be a source of information for architects when designing geodesic domes. Additionally, this paper can be helpful in assessing the effects of earthquakes on lightweight structures.

## Figures and Tables

**Table 1:** Division of the analysed geodesic domes into four groups of struts.

Groups of Struts	
2888-Hedron (Method 1)	2904-Hedron (Method 2)
RO 70.0 × 8.0	RO 70.0 × 7.1
RO 63.5 × 8.8	RO 63.5 × 8.0
RO 44.5 × 5.6	RO 57.0 × 5.6
RO 44.5 × 3.6	RO 51.0 × 3.2

**Table 2:** Results from numerical analysis.

Seismic Records								
	Ancona (Italia)		Denizil (Turkey)		Friuli (Italia)		Kilini (Greece)	
Method								
Direction	1	2	1	2	1	2	1	2
	Model I	Model II	Model III	Model IV	Model V	Model VI	Model VII	Model VIII
Displacement (mm)								
x	17.4	6.8	8.3	6.6	3.9	2.3	−1.9	1.1
y	23.4	−7.6	13.3	−6.9	3.6	2.1	1.1	0.9
z	10.2	4.5	6.7	5.6	1.7	0.9	1.0	0.6
Axial forces (kN)								
	−69.54	47.01	40.54	37.63	12.82	13.31	5.56	−6.76
Velocity (m/s)								
x	0.99	0.42	0.50	0.44	0.18	0.09	0.12	0.07
y	1.34	0.45	0.79	0.42	0.23	0.10	0.07	0.05
z	0.62	0.32	0.44	0.36	0.10	0.07	0.06	0.05
Acceleration (m/s^2^)								
x	64.14	29.34	33.77	26.48	10.67	5.88	8.90	4.78
y	81.71	30.19	55.78	27.44	13.50	6.66	4.89	3.49
z	39.21	32.42	28.06	−34.35	6.26	6.01	4.51	3.79

## Data Availability

The data presented in this study are available within the article.
