# Correction: Bysiec et al. Numerical Analysis of Steel Geodesic Dome under Seismic Excitations. *Materials* 2021, *14*, 4493

**DOI:** 10.3390/ma17010249

**Published:** 2024-01-03

**Authors:** Dominika Bysiec, Tomasz Maleska

**Affiliations:** Faculty of Civil Engineering and Architecture, Opole University of Technology, 45-758 Opole, Poland; d.bysiec@po.edu.pl

In the original publication [1], Figure 1 has been removed. The remaining figures have been renumbered in order from number 1. The authors state that the scientific conclusions are unaffected. This correction was approved by the Academic Editor. The original publication has also been updated.
